# Supplementary material for: Metabolomics of Plasma in XLH Patients with Arterial Hypertension: New Insights into the Underlying Mechanisms
Source: Int J Mol Sci. 2024 Mar 21;25(6):3545. doi: 10.3390/ijms25063545 (PMC10971556; doi:10.3390/ijms25063545)
Supplement: Supplementary file 1 [file ijms-25-03545-s001.zip › ijms-2903317-supplementary.pdf]

Supplementary. There was no correlation observed between acetylcarnitine, pyruvate, butyric acid, or ethanolamine and the levels of PTH, vitamin D, phosphorus, or FGF23. Figure with results of this analysis.

FGF-23

Model Summary

Model	R	R Square	Adjusted R Square	Std. Error of the Estimate	Change Statistics				
					R Square Change	F Change	df1	df2	Sig. F Change
1	,289[a]	,083	,032	244,53518	,083	1,635	1	18	,217

a. Predictors: (Constant), Acetylcarnitine

Model Summary

Model	R	R Square	Adjusted R Square	Std. Error of the Estimate	Change Statistics				
					R Square Change	F Change	df1	df2	Sig. F Change
1	,325[a]	,106	,056	241,49939	,106	2,132	1	18	,162

a. Predictors: (Constant), Pyruvate

Model Summary

Model	R	R Square	Adjusted R Square	Std. Error of the Estimate	Change Statistics				
					R Square Change	F Change	df1	df2	Sig. F Change
1	,154[a]	,024	-,031	252,36417	,024	,435	1	18	,518

a. Predictors: (Constant), Butyricacid

Model Summary

Model	R	R Square	Adjusted R Square	Std. Error of the Estimate	Change Statistics				
					R Square Change	F Change	df1	df2	Sig. F Change
1	,160[a]	,026	-,029	252,11115	,026	,472	1	18	,501

a. Predictors: (Constant), Ethanolamine

Phosphate

Model Summary

Model	R	R Square	Adjusted R Square	Std. Error of the Estimate	Change Statistics				
					R Square Change	F Change	df1	df2	Sig. F Change
1	,559[a]	,313	,275	,42302	,313	8,198	1	18	,010

a. Predictors: (Constant), Ethanolamine

Model Summary

Model	R	R Square	Adjusted R Square	Std. Error of the Estimate	Change Statistics				
					R Square Change	F Change	df1	df2	Sig. F Change
1	,276[a]	,076	,025	,49053	,076	1,483	1	18	,239

a. Predictors: (Constant), Butyricacid

Model Summary

Model	R	R Square	Adjusted R Square	Std. Error of the Estimate	Change Statistics				
					R Square Change	F Change	df1	df2	Sig. F Change
1	,232[a]	,054	,001	,49645	,054	1,021	1	18	,326

a. Predictors: (Constant), Acetylcarnitine

Model Summary

Model	R	R Square	Adjusted R Square	Std. Error of the Estimate	Change Statistics				
					R Square Change	F Change	df1	df2	Sig. F Change
1	,059[a]	,003	-,052	,50946	,003	,062	1	18	,806

a. Predictors: (Constant), Pyruvate

Vitamin D

Model Summary

Model	R	R Square	Adjusted R Square	Std. Error of the Estimate	Change Statistics				
					R Square Change	F Change	df1	df2	Sig. F Change
1	,149[a]	,022	-,035	9,61240	,022	,384	1	17	,544

a. Predictors: (Constant), Butyricacid

Model Summary

Model	R	R Square	Adjusted R Square	Std. Error of the Estimate	Change Statistics				
					R Square Change	F Change	df1	df2	Sig. F Change
1	,223[a]	,050	-,006	9,47491	,050	,892	1	17	,358

a. Predictors: (Constant), Ethanolamine

Model Summary

Model	R	R Square	Adjusted R Square	Std. Error of the Estimate	Change Statistics				
					R Square Change	F Change	df1	df2	Sig. F Change
1	,263[a]	,069	,015	9,37751	,069	1,266	1	17	,276

a. Predictors: (Constant), Acetylcarnitine

Model Summary

Model	R	R Square	Adjusted R Square	Std. Error of the Estimate	Change Statistics				
					R Square Change	F Change	df1	df2	Sig. F Change
1	,141[a]	,020	-,038	9,62288	,020	,346	1	17	,564

a. Predictors: (Constant), Pyruvate

PTH

Model Summary

Model	R	R Square	Adjusted R Square	Std. Error of the Estimate	Change Statistics				
					R Square Change	F Change	df1	df2	Sig. F Change
1	,072[a]	,005	-,050	50,08101	,005	,094	1	18	,763

a. Predictors: (Constant), Butyricacid

Model Summary

Model	R	R Square	Adjusted R Square	Std. Error of the Estimate	Change Statistics				
					R Square Change	F Change	df1	df2	Sig. F Change
1	,349[a]	,122	,073	47,05728	,122	2,494	1	18	,132

a. Predictors: (Constant), Ethanolamine

Model Summary

Model	R	R Square	Adjusted R Square	Std. Error of the Estimate	Change Statistics				
					R Square Change	F Change	df1	df2	Sig. F Change
1	,231[a]	,053	,001	48,85593	,053	1,013	1	18	,328

a. Predictors: (Constant), Acetilcarnitine

Model Summary

Model	R	R Square	Adjusted R Square	Std. Error of the Estimate	Change Statistics				
					R Square Change	F Change	df1	df2	Sig. F Change
1	,069[a]	,005	-,051	50,09255	,005	,086	1	18	,773

a. Predictors: (Constant), Pyruvate
